# New Insights in Transcriptional Regulation of the Ethylene Response in *Arabidopsis*

**DOI:** 10.3389/fpls.2019.00790

**Published:** 2019-06-18

**Authors:** Likai Wang, Hong Qiao

**Affiliations:** ^1^Institute for Cellular and Molecular Biology, The University of Texas at Austin, Austin, TX, United States; ^2^Department of Molecular Biosciences, The University of Texas at Austin, Austin, TX, United States

**Keywords:** ethylene, transcription regulation, *Arabidopsis*, histone, hormone

## Abstract

As any living organisms, plants must respond to a wide variety of environmental stimuli. Plant hormones regulate almost all aspects of plant growth and development. Among all the plant hormones, ethylene is the only gaseous plant hormone that plays pleiotropic roles in plant growth, plant development and stress responses. Transcription regulation is one main mechanism by which a cell orchestrates gene activity. This control allows the cell or organism to respond to a variety of intra- and extracellular signals and thus mount a response. Here we review the progress of transcription regulation in the ethylene response.

## Transcriptional Regulation in the Ethylene Response

Like all living organisms, plants must respond to a wide variety of environmental stimuli. Plant hormones, produced in response to environmental stimuli, regulate almost all aspects of plant growth and development. Ethylene is a gaseous plant hormone that plays pleiotropic roles in plant growth, plant development, and stress responses. Histone acetylation, which is modulated through ethylene-mediated signaling, regulates dynamic changes in chromatin structure that result in transcriptional regulation in responses to ethylene.

Ethylene is perceived by a family of receptors bound to the endoplasmic reticulum (ER) membrane (Chang et al., 1993; Bleecker et al., 1998; Hua and Meyerowitz, 1998; Hua et al., 1998; Sakai et al., 1998). Each receptor binds ethylene via a copper cofactor that is provided by the copper transporter *RESPONSIVE-TO-ANTAGONIST 1* (RAN1)(Hirayama et al., 1999). In the absence of ethylene, ethylene receptor ETHYLENE RECEPTOR 1 (ETR1) interacts with CONSTITUTIVE TRIPLE RESPONSE 1 (CTR1), a downstream negative regulator of ethylene signaling (Chang et al., 1993; Kieber et al., 1993; Gao et al., 2003; Shakeel et al., 2015). CTR1 is a protein kinase that phosphorylates ETHYLENE INSENSITIVE 2 (EIN2), a key positive regulator of ethylene signaling (Alonso et al., 1999; Ju et al., 2012), preventing the ethylene response. In addition, in the absence of ethylene, EIN2 protein levels are regulated by EIN2 TARGETING PROTEIN 1 and 2 (ETP1/2) via ubiquitin/proteasome-mediated degradation (Qiao et al., 2009).

In the presence of ethylene both ethylene receptors and CTR1 are inactivated, and the C-terminal end of EIN2 is dephosphorylated and cleaved by unknown mechanisms. The cleaved C-terminal end of EIN2 translocates to the nucleus (Qiao et al., 2012; Wen et al., 2012; Ju et al., 2015) where it facilitates the acetylation of histone 3 at K14 and K23 (H3K14 and H3K23, respectively) to regulate ETHYLENE INSENSITIVE 3 (EIN3) and ETHYLENE INSENSITIVE 3 LIKE1 (EIL1) – dependent transcriptional regulation (Zhang et al., 2016). The cleaved EIN2 C-terminal end also translocates into the P-body through associating with 3′UTRs of EIN3 BINDING F-BOX1 (EBF1) and EBF2, further repressing their translation (Guo and Ecker, 2003). EBF1 and EBF2 in turn stabilize EIN3 and EIL1, resulting in activation of EIN3- and EIL1-dependent transcription and the activation of an ethylene response (Li et al., 2015; Merchante et al., 2015).

Both genetic and molecular studies have demonstrated that EIN3 and EIL1 are positive regulators that are necessary and sufficient for the ethylene response (Chao et al., 1997; Guo and Ecker, 2003; Chang et al., 2013). The *EIN3* gene encodes a nuclear-localized protein that is essential to the response to ethylene (Chao et al., 1997). In the absence of EIN3, plants are partially insensitive to ethylene both at the morphological and molecular levels (Chao et al., 1997; Guo and Ecker, 2003). The EIN3 binding motif was identified after analysis of the promoters of the genes that are highly up-regulated by ethylene and followed by validation using an electrophoresis mobility shift assay (EMSA) (Ohme-Takagi and Shinshi, 1990; Eyal et al., 1993; Meller et al., 1993; Sessa et al., 1995; Shinshi et al., 1995; Sato et al., 1996; Solano et al., 1998). Using the EMSA assay, EIN3 was shown to form a homodimer in the presence of DNA *in vitro* (Solano et al., 1998). However, whether the homodimer is formed *in vivo* and whether the homodimer is required for EIN3 to function in the ethylene signaling are unknown. A number of transcription factors are known to form homodimers or heterodimers, which have different specificities and affinities for certain DNA motifs (Funnell and Crossley, 2012). Finding out whether the dimerization is necessary for EIN3′s function *in vivo* will be an interesting question in the transcriptional regulation of the ethylene response.

To explore the transcription regulation in response to ethylene, Chang et al characterized the dynamic ethylene transcriptional response by identifying targets of EIN3, the master regulator of the ethylene signaling pathway, using chromatin immunoprecipitation sequencing and transcript sequencing during a time course of ethylene treatment (Chang et al., 2013). They found that the number of genes bound by EIN3 does not change significantly in response to ethylene. The amount of EIN3 bound increases upon ethylene treatment, and the expression of most of EIN3-bound genes is up regulated by ethylene, which is consistent with a role of EIN3 as a transcriptional activator. Chang et al. also analyzed the sequences of EIN3-bound regions identified by ChIP-seq (Ohme-Takagi and Shinshi, 1990; Eyal et al., 1993; Meller et al., 1993; Sessa et al., 1995; Shinshi et al., 1995; Sato et al., 1996; Solano et al., 1998). A motif similar to that previously identified in the promoter regions of ethylene up-regulated genes was present in EIN3-bound regions (Ohme-Takagi and Shinshi, 1990; Eyal et al., 1993; Meller et al., 1993; Sessa et al., 1995; Shinshi et al., 1995; Sato et al., 1996; Solano et al., 1998). Intriguingly, Chang et al. also found that ethylene-induced transcription occurs in temporal waves that were regulated by EIN3 with the potentially distinct layers of transcriptional control (Chang et al., 2013). EIN3 binding was found to modulate a multitude of downstream transcriptional cascades, including a major feedback regulatory circuitry of the ethylene signaling pathway, as well as most of the hormone-mediated growth response pathways, which indicates that network-level feedback regulation results in overall system control and homeostasis (Chang et al., 2013). This type of study can be applied to identify novel components in signaling pathways (Rosenfeld et al., 2002; Amit et al., 2007; Tsang et al., 2007; Avraham and Yarden, 2011; Feng et al., 2011; Yosef and Regev, 2011).

**FIGURE 1:**
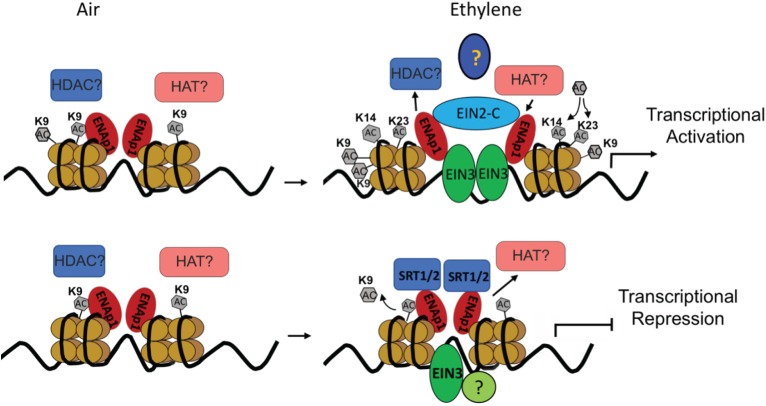
Diagram to illustrate the transcription regulation in ethylene response. Upper panel: in the absence of ethylene, no ethylene regulated transcription regulation; In the presence of ethylene, accumulated EIN3 interacts with ENAP1 and EIN2 c-terminus, which elevates histone acetylation of H3K14Ac and H3K23Ac through an interaction with unidentified histone acetyltransferases to activate EIN3 dependent transcriptional activation. Lower panel: In the absence of ethylene, ethylene down-regulated genes are transcribed; In the presence of ethylene, histone deacetylase SRT1 and SRT2 are recruited to target genes to keep a low level of H3K9Ac, resulting in the targets for transcriptional repression.

Although the transcriptional activation has been the main focus in the ethylene response, transcriptome analysis in Chang’s study clearly showed that nearly 50% of ethylene-altered genes are down regulated and that a subset of ethylene-repressed genes are bound by EIN3 (Chang et al., 2013). Notably, most of the genes are down regulated by ethylene within 1 h of treatment. This result strongly suggests that transcriptional repression plays a critical role in early ethylene response. Interestingly, a recent study from the Qiao lab showed that transcriptional repression dose plays important roles in ethylene response. We identified two histone deacetylases (HDACs) SIRTUIN 1 and 2 (SRT1 and SRT2) that regulate ethylene-repressed genes (Zhang et al., 2018). Notably the study found that SRT2 binds the target promoter regions to inhibit acetylation of histone 3 at K9 (H3K9Ac), repressing gene expression in response to ethylene (Figure 1).

Transcriptional repression by chromatin modification is one of the principal mechanisms employed by eukaryotic active repressors (Thiel et al., 2004; Kagale and Rozwadowski, 2011). The importance of HDACs in transcriptional repression during plant growth and development has been well established (Song et al., 2005; Li et al., 2017). For example, in *Arabidopsis*, the EAR motif containing class II ETHYLENE RESPONSIVE ELEMENT BINDING FACTORS (ERFs), such as ERF3 and ERF4, which are known to function as active repressors *in vitro* and *in vivo*, have been shown to physically interact with AtSAP18, which in turn interacts and forms a repression complex with AtHDA19 (Fujimoto et al., 2000; Ohta et al., 2001; McGrath et al., 2005; Song and Galbraith, 2006). AtERF7, another EAR motif-containing class II ERF protein, is also known to recruit AtHDA19 via a physical interaction with AtSIN3 (Song et al., 2005). *In planta*, coexpression of AtERF3, AtSAP18, and AtHDA19 or AtERF7, AtSIN3, and AtHDA19 results in greater transcriptional repression of reporter genes as compared to when these proteins are expressed alone suggesting a role for AtSAP18, AtSIN3, and AtHDA19 in ERF-mediated transcriptional repression possibly via histone deacetylation (Song et al., 2005; Song and Galbraith, 2006). Yet, whether EAR containing proteins are also required for SRT1 and SRT2 mediated transcriptional repression in response to ethylene is unknown (Zhang et al., 2018). It also remains unclear whether the ethylene response has a molecular mechanism of transcriptional repression similar to that induced by other plant hormones. Exploring the molecular mechanism of transcriptional repression will provide more insight into ethylene signaling and ethylene response.

## Histone Acetylation in Ethylene-Mediated Transcriptional Regulation

In eukaryotes, the binding of transcription factors is mainly determined by chromatin structure, namely the state of the genome’s packaging with specific structural proteins, mainly histones. Chromatin undergoes different dynamics structure changes, further influences transcription factor binding. Among all the regulations, histone acetylation results in a switch between repressive and permissive chromatin. In general, acetylation neutralizes the positive charges of lysine residues and decreases the interaction between histone and DNA, leading to a more relaxed chromatin structure, which is associated with transcriptional activation. In contrast, deacetylation induces a compact chromatin structure, which is associated with transcriptional repression (Fletcher and Hansen, 1996; Steger and Workman, 1996; Luger and Richmond, 1998).

A Number of studies have shown a tight link between histone acetylation and plant hormone responses (Zhu, 2010). In the studies of ethylene signaling, authors found that GENERAL CONTROL NON-REPRESSED PROTEIN 5 (GCN5), which belongs to a family of histone acetyl transferases (HATs), promotes transcriptional activation (Brownell and Allis, 1996; Grant et al., 1997; Bian et al., 2011; Weake and Workman, 2012; Ryu et al., 2014). The *Arabidopsis gcn5* mutant shows hypersensitivity to ethylene treatment. In the *gcn5* mutant, the histone acetylation at H3K9 and H3K14 in the promoter regions of ethylene response genes is elevated, and the elevation is associated with the up-regulation of gene expression (Poulios and Vlachonasios, 2016). In *Arabidopsis*, the HAC family, which are the homologs of CREB-binding protein (CBP) and p300, the mammalian family of HAT domain containing transcriptional coactivators, play pleotropic roles in plant growth and development (Pandey et al., 2002; Li et al., 2014). The *hac1hac5* double mutant was found to have a constitutive triple response phenotype (Pandey et al., 2002; Li et al., 2014). It was expected that gene expression would be down regulated in the *hac1hac5* double mutant due to the reduction of histone acetylation levels; however, similar to that in *gcn5* mutant, the downstream ethylene responsive genes are elevated in *hac1hac5* double mutant (Li et al., 2014), suggesting an indirect regulation of ethylene responsive genes by HAC1 and HAC5.

Expression of two HDACs, *HAD6* and *HDA19*, are specifically elevated by ethylene treatment. The expression of ethylene responsive gene *ERF1* is anti-correlated with the levels of histone H3 acetylation in *35S*:*HDA19* transgenic plants, showing that HDA19 indirectly influences *ERF1* gene expression (Zhou et al., 2005). It is possible that HDA19 induces ERF1 expression by preventing binding of an unknown transcription repressor that regulates ERF1 expression (Zhou et al., 2005). JAZ proteins recruit HDA6 to deacetylate histones and obstruct the chromatin binding of EIN3/EIL1, therefore repressing EIN3/EIL1-dependent transcription and inhibiting jasmonic acid-mediated signaling (Zhu et al., 2011). This provides evidence that histone acetylation regulates EIN3 target genes.

H3K14Ac and H3K23Ac, but not H3K9Ac, H3K18Ac, or H3K27Ac, are elevated by ethylene treatment at the molecular level (Zhang et al., 2016; Yoon et al., 2017). Interestingly, even though the levels of H3K9Ac are not regulated by ethylene, the levels of H3K9Ac in the promoters of ethylene up-regulated genes are higher than that in those ethylene down-regulated genes both with and without ethylene treatments (Zhang et al., 2016; Yoon et al., 2017). Presumably, H3K9Ac is a pre-existing mark that labels genes regulated by ethylene, whereas the elevation of H3K14Ac and H3K23Ac is positively associated with gene activation. Most importantly, the ethylene-induced change of histone acetylation is EIN2 dependent. However, EIN2 is not a histone or a DNA binding protein, and the biochemical function of EIN2-C remains unknown. Yeast two-hybrid screening and ChIP-re-ChIP suggests that EIN2-C is associated with histones at least in part through EIN2 NUCLEAR ASSOCIATED PROTEIN 1 (ENAP1), which has histone binding activity (Figure 1; Zhang et al., 2017). It is possible that EIN2-C is a scaffolding protein that is important for the formation of HAT-containing protein complexes in response to ethylene. Identification of HAT or HDAC that functions in cooperation with EIN2-C would validate this assumption. In contrast to transcriptional activation, histone acetylation of H3K9Ac was found to be involved in the transcriptional repression, and the regulation is partially mediated by histone deacetylase SRT1 and SRT2 (Figure 1).

Taken together, currently available data suggest that in the absence of ethylene ENAP1 binds to histones to keep chromatin in a relaxed state poised for a rapid response to ethylene (Figure 1). In the presence of ethylene, EIN2-C is translocated to the nucleus where it interacts with ENAP1 and potentially HATs resulting in histone acetylation. This causes an uncompacting of chromatin, resulting in more EIN3 binding to target genes and ultimately transcription activation (Figure 1). It is not known how the histone acetylation targets are determined in the presence of ethylene. Zhang et al. showed that EIN3 is partially required for the ethylene-induced elevation of H3K14Ac and H3K23Ac (Pandey et al., 2002), suggesting that EIN3 might mark histone acetylation targets. Multi-protein assemblies have been shown to determine the substrate specificities and targeting of integral HAT subunits. The molecular mechanism of how EIN3, ENAP1, and EIN2-C coordinate to integrate the histone acetylation and transcription regulation remains to be elucidated. Beside histone acetylation regulation in transcriptional activation, histone acetylation has been shown to be involved in transcriptional repression in ethylene response. As mentioned above, SRT1 and SRT2 mediate transcriptional repression that requires a low level of H3K9Ac (Yosef and Regev, 2011). How the H3K9Ac levels are determined in the desired targets in the first place is an interesting and important question that needs to be addressed.

## Concluding Remarks and Future Perspectives

Plants must respond accurately and quickly to hormones, and this necessitates a flexible and rapid way to control gene expression. The acetylation of histone tails by HATs neutralizes positive charges on these proteins that would otherwise interact with negatively charged DNA, facilitating nucleosome unwrapping for rapid transcription activation. How plants utilize a limited number of HATs and HDACs to specifically regulate responses to different hormones is largely unknown. Presumably, the specificity relies on the partners of HATs and HDACs. Identification of the HAT- and HDAC-containing complexes upon ethylene treatment will reveal details of the molecular mechanisms that underlie the ethylene response. Recent studies have clearly shown that different tissues respond to plant hormones differently (Garg et al., 2012; Pattison et al., 2015; Raines et al., 2016). Most available data on histone acetylation induced by plant hormones come from analyses of the whole plant. Studies of histone acetylation in individual tissues and in different cell types will provide more detailed insight into how histone acetylation controls responses to plant hormones. Transcription factor binding in eukaryotes is highly dependent on the context of binding sites on chromatin, but little is known about how EIN3 determines histone acetylation sites in target genes. A more complete understanding of the molecular mechanism of determination of transcriptional activation and transcriptional repression during the ethylene response will facilitate development of methods to improve crop production.

## Author Contributions

Both authors listed have made a substantial, direct and intellectual contribution to the work, and approved it for publication.

## Conflict of Interest Statement

The authors declare that the research was conducted in the absence of any commercial or financial relationships that could be construed as a potential conflict of interest.
